# Crystal structure of aqua­bis­[2-(1*H*-benzimidazol-2-yl-κ*N* ^3^)aniline-κ*N*]zinc dinitrate

**DOI:** 10.1107/S2056989015004636

**Published:** 2015-03-14

**Authors:** Yongtae Kim, Sung Kwon Kang

**Affiliations:** a Department of Chemistry, Chungnam National University, Daejeon 305-764, Republic of Korea

**Keywords:** crystal structure, zinc complex, benz­imidazole, hydrogen bonding

## Abstract

The cation of the complex title salt, [Zn(C_13_H_11_N_3_)_2_(H_2_O)](NO_3_)_2_, lies about a twofold rotation axis, which passes through the Zn^II^ atom and the O atom of the aqua ligand. The Zn^II^ atom adopts a distorted trigonal–bipyramidal geometry defined by two N atoms in axial positions [angle = 166.24 (7)°], and two N and one O atom in the equatorial plane [range of angles: 115.17 (7)–122.42 (3)°]. The dihedral angle between the imidazole and aniline rings is 23.86 (5)°. In the crystal, N—H⋯O and O—H⋯O hydrogen bonds link the components into a three-dimensional network.

## Related literature   

For the synthesis of the title complex and derivatives, see: Esparza-Ruiz *et al.* (2011 ▸); Eltayeb *et al.* (2011 ▸). For background to benz­imidazoles and their applications, see: Chassaing *et al.* (2008 ▸); Podunavac-Kuzmonovic *et al.* (1999 ▸); Sánchez-Guadarrama *et al.* (2009 ▸); Xue *et al.* (2011 ▸).
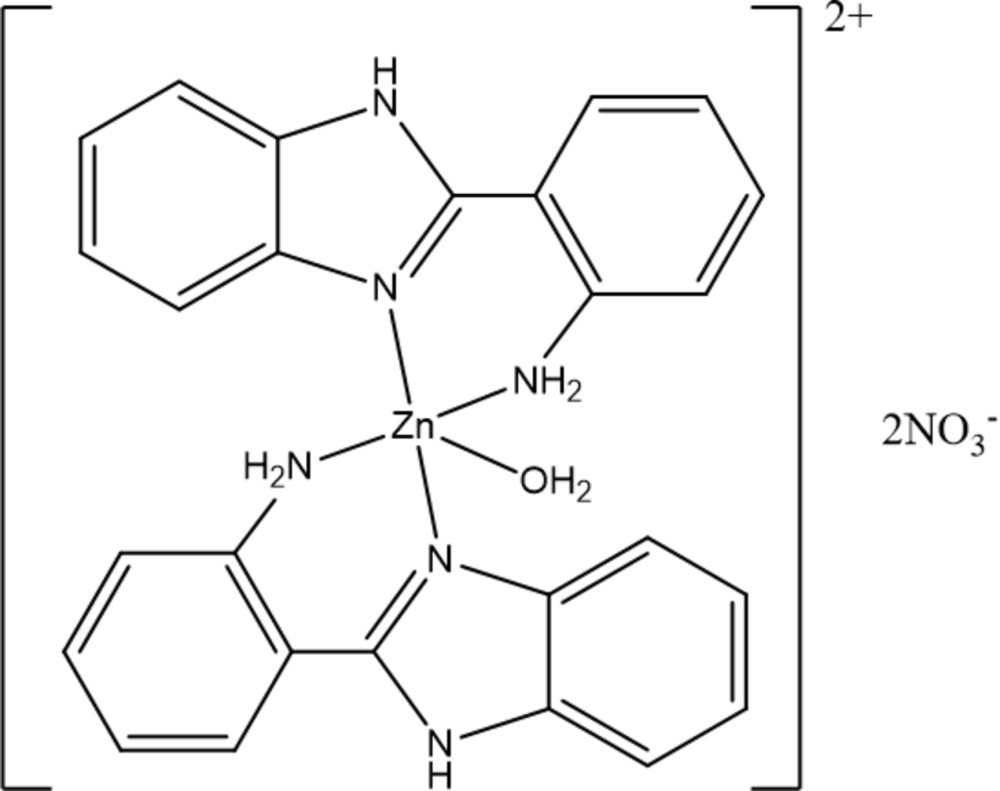


## Experimental   

### Crystal data  

[Zn(C_13_H_11_N_3_)_2_(H_2_O)](NO_3_)_2_
*M* *_r_* = 625.9
Monoclinic, 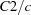

*a* = 16.2892 (9) Å
*b* = 15.0782 (8) Å
*c* = 11.6840 (6) Å
β = 110.0178 (8)°
*V* = 2696.4 (2) Å^3^
*Z* = 4
Mo *K*α radiation
μ = 0.97 mm^−1^
*T* = 296 K
0.21 × 0.20 × 0.18 mm

### Data collection  

Bruker SMART CCD area-detector diffractometer
Absorption correction: multi-scan (*SADABS*; Bruker, 2002 ▸) *T*_min_ = 0.546, *T*_max_ = 0.726
13558 measured reflections
3347 independent reflections
3007 reflections with *I* > 2σ(*I*)
*R*_int_ = 0.020

### Refinement  

*R*[*F* ^2^ > 2σ(*F* ^2^)] = 0.027
*wR*(*F* ^2^) = 0.078
*S* = 1.06
3347 reflections
207 parameters
H atoms treated by a mixture of independent and constrained refinement
Δρ_max_ = 0.35 e Å^−3^
Δρ_min_ = −0.20 e Å^−3^

### 

Data collection: *SMART* (Bruker, 2002 ▸); cell refinement: *SAINT* (Bruker, 2002 ▸); data reduction: *SAINT*; program(s) used to solve structure: *SHELXS2013* (Sheldrick, 2008 ▸); program(s) used to refine structure: *SHELXL2013* (Sheldrick, 2015 ▸); molecular graphics: *ORTEP-3 for Windows* (Farrugia, 2012 ▸); software used to prepare material for publication: *WinGX* (Farrugia, 2012 ▸).

## Supplementary Material

Crystal structure: contains datablock(s) global, I. DOI: 10.1107/S2056989015004636/tk5361sup1.cif

Structure factors: contains datablock(s) I. DOI: 10.1107/S2056989015004636/tk5361Isup2.hkl

**Fig. 1.**
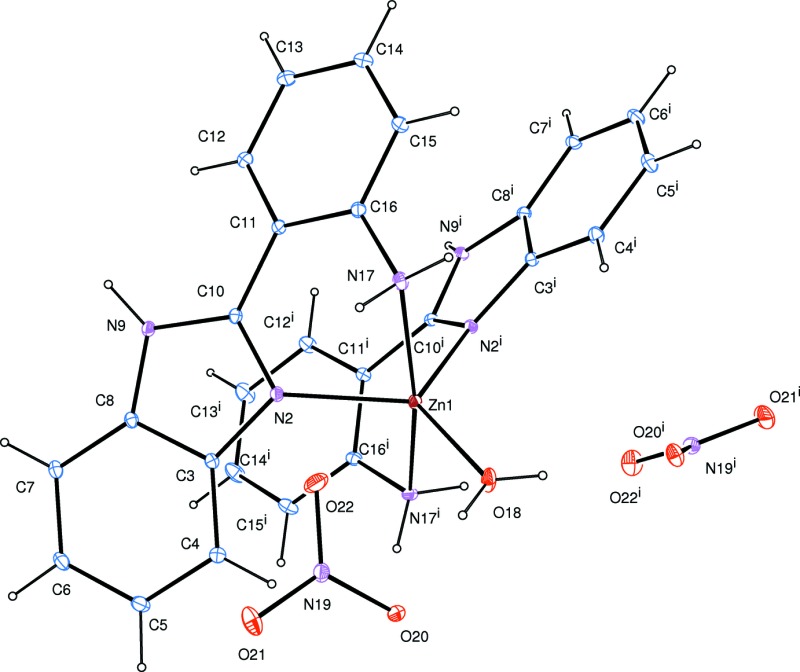
Molecular structure of the title complex, showing the atom-numbering scheme and 30% probability ellipsoids. [Symmetry code: (i): -x + 1, y, -z + 1/2]

Click here for additional data file.x y z . DOI: 10.1107/S2056989015004636/tk5361fig1.tif
Mol­ecular structure of the title complex, showing the atom-numbering scheme and 30% probability ellipsoids. [Symmetry code: (i): −*x* + 1, *y*, −*z* + 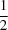]

**Fig. 2.**
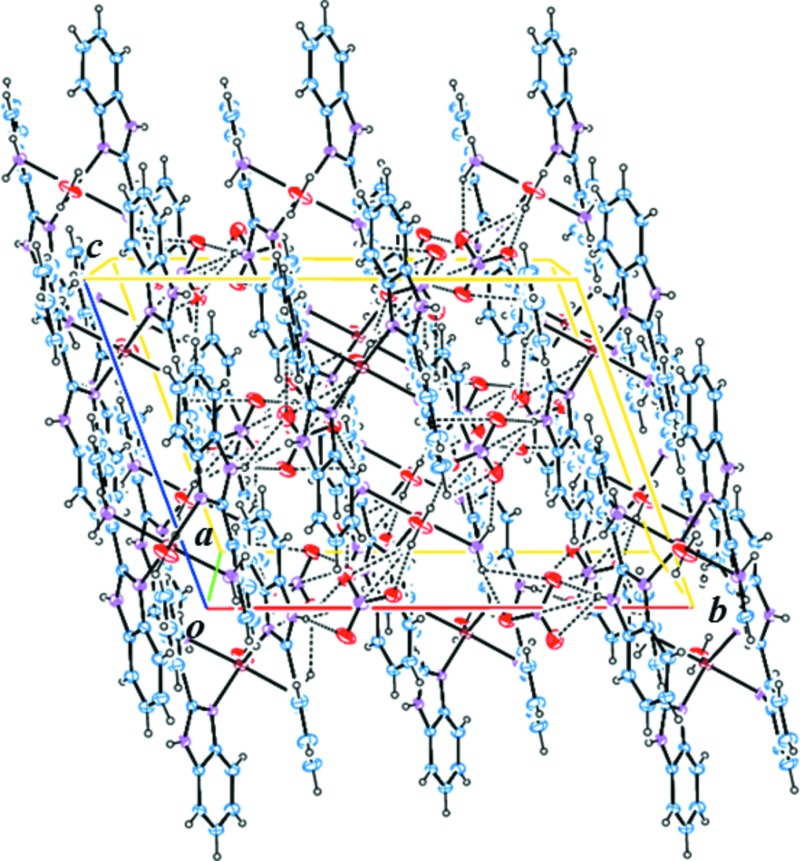
Part of the crystal structure of the title complex, showing the 3-D network of molecules linked by intermolecular N—H···O and O—H···O hydrogen bonds (dashed lines).

. DOI: 10.1107/S2056989015004636/tk5361fig2.tif
Part of the crystal structure of the title complex, showing the 3-D network of mol­ecules linked by inter­molecular N—H⋯O and O—H⋯O hydrogen bonds (dashed lines).

CCDC reference: 1052527

Additional supporting information:  crystallographic information; 3D view; checkCIF report

## Figures and Tables

**Table 1:** Hydrogen-bond geometry (, )

*D*H*A*	*D*H	H*A*	*D* *A*	*D*H*A*
N9H9O21^i^	0.74(2)	2.51(2)	3.248(2)	173(2)
N9H9O22^i^	0.74(2)	2.37(2)	2.944(2)	134.7(19)
N17H17*A*O20^ii^	0.86(2)	2.14(2)	2.9937(17)	169(2)
O18H18O20	0.77(2)	1.92(2)	2.6897(14)	175(2)
O18H18O22	0.77(2)	2.50(2)	3.0345(16)	128(2)

Symmetry codes: (i) 
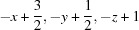
; (ii) 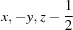.

## supplementary crystallographic information

### S1. Structural commentary

The heterocycles azole and benzazole have been of inter­est in several important functions in biological systems (Esparza-Ruiz *et al.*, 2011; Eltayeb *et al.*, 2011). benzimidazole compounds show a variety of biological properties such as inhibitory activities against enteroviruses and anti­bacterials (Xue *et al.*, 2011; Chassaing *et al.*, 2008; Sánchez-Guadarrama *et al.*, 2009). Transition metal complexes with benzimidazole derivatives have been studied as models of some important biological molecules (Podunavac-Kuzmonovic *et al.*, 1999). Motivated by these studies, the title complex has been synthesized and characterized by X-ray crystallography.

In the title complex, the Zn^II^ atom lies on a two-fold axis and is coordinated by one O atom and four N atoms of two bidentate imidazole­aniline ligands, forming a distorted trigonal bipyrdmidal geometry. The axial Zn1—N17 bond distance of 2.2147 (17) Å is longer than the equatorial Zn1—N2 distance of 2.0421 (11) Å. The N17—Zn1—N17^i^ axial angle is 166.24 (7)°, and the angles of two N and one O atom in the equatorial plane is within the range of 115.17 (7) and 122.42 (3)°. The dihedral angle between the imidazole and aniline rings in the coordinated bidentate ligand is 23.86 (5)°. In the crystal, inter­molecular N—H···O and O—H···O hydrogen bonds link the molecules into a three-dimensional network.

### S2. Synthesis and crystallization

To a stirred solution of 2-(2-amino­phenyl)-1*H*-benzimidazole (0.188 g, 0.9 mmol) in EtOH (20 ml) was added a solution of zinc nitrate hexahydrate (0.089 g, 0.3 mmol) in EtOH (10 mL) at 60 °. After 24 h of reflux, the color of solution turned yellow. The product was isolated as a pale yellow powder by removing the solvent. Yellow single crystals of the title complex were obtained from its methanol solution by slow evaporation of the solvent at room temperature within several days.

### S3. Refinement

H atoms on NH, NH_2_, and OH_2_ groups were located in a difference Fourier map and refined freely [refined N—H distances = 0.74 (2)–0.87 (2), O—H = 0.77 (2)Å]. Other H atoms were positioned geometrically and refined using riding model, with d(C—H) = 0.93 Å, and with U_iso_(H) = 1.2U_eq_(C).

### Crystal data

**Table:**

[Zn(C_13_H_11_N_3_)_2_(H_2_O)](NO_3_)_2_	*F*(000) = 1288
*M**_r_* = 625.9	*D*_x_ = 1.542 Mg m^−^^3^
Monoclinic, *C*2/*c*	Mo *K*α radiation, λ = 0.71073 Å
Hall symbol: -C 2yc	Cell parameters from 6738 reflections
*a* = 16.2892 (9) Å	θ = 2.7–28.0°
*b* = 15.0782 (8) Å	µ = 0.97 mm^−^^1^
*c* = 11.6840 (6) Å	*T* = 296 K
β = 110.0178 (8)°	Block, yellow
*V* = 2696.4 (2) Å^3^	0.21 × 0.2 × 0.18 mm
*Z* = 4	

### Data collection

**Table:**

Bruker SMART CCD area-detector diffractometer	3007 reflections with *I* > 2σ(*I*)
Radiation source: fine-focus sealed tube	*R*_int_ = 0.020
φ and ω scans	θ_max_ = 28.3°, θ_min_ = 1.9°
Absorption correction: multi-scan (*SADABS*; Bruker, 2002)	*h* = −21→21
*T*_min_ = 0.546, *T*_max_ = 0.726	*k* = −19→20
13558 measured reflections	*l* = −15→11
3347 independent reflections	

### Refinement

**Table:**

Refinement on *F*^2^	0 restraints
Least-squares matrix: full	Hydrogen site location: mixed
*R*[*F*^2^ > 2σ(*F*^2^)] = 0.027	H atoms treated by a mixture of independent and constrained refinement
*wR*(*F*^2^) = 0.078	*w* = 1/[σ^2^(*F*_o_^2^) + (0.0439*P*)^2^ + 1.0441*P*] where *P* = (*F*_o_^2^ + 2*F*_c_^2^)/3
*S* = 1.06	(Δ/σ)_max_ < 0.001
3347 reflections	Δρ_max_ = 0.35 e Å^−^^3^
207 parameters	Δρ_min_ = −0.20 e Å^−^^3^

### Special details

**Table:**

Geometry. All e.s.d.'s (except the e.s.d. in the dihedral angle between two l.s. planes) are estimated using the full covariance matrix. The cell e.s.d.'s are taken into account individually in the estimation of e.s.d.'s in distances, angles and torsion angles; correlations between e.s.d.'s in cell parameters are only used when they are defined by crystal symmetry. An approximate (isotropic) treatment of cell e.s.d.'s is used for estimating e.s.d.'s involving l.s. planes.

### Fractional atomic coordinates and isotropic or equivalent isotropic displacement parameters (Å^2^)

**Table:**

	*x*	*y*	*z*	*U*_iso_*/*U*_eq_	
Zn1	0.5	0.15270 (2)	0.25	0.03249 (9)	
N2	0.59261 (7)	0.22530 (8)	0.37818 (10)	0.0317 (2)	
C3	0.61501 (9)	0.22740 (10)	0.50420 (12)	0.0331 (3)	
C4	0.60111 (11)	0.16542 (11)	0.58370 (15)	0.0436 (4)	
H4	0.5726	0.1121	0.5556	0.052*	
C5	0.63145 (12)	0.18640 (14)	0.70620 (15)	0.0522 (4)	
H5	0.6242	0.1458	0.7619	0.063*	
C6	0.67293 (11)	0.26740 (13)	0.74848 (14)	0.0499 (4)	
H6	0.6914	0.2797	0.8314	0.06*	
C7	0.68716 (10)	0.32919 (11)	0.67076 (14)	0.0413 (3)	
H7	0.7143	0.3831	0.6989	0.05*	
C8	0.65875 (9)	0.30678 (10)	0.54755 (12)	0.0338 (3)	
N9	0.66310 (9)	0.35081 (9)	0.44630 (12)	0.0348 (3)	
H9	0.6898 (13)	0.3906 (14)	0.4450 (18)	0.045 (5)*	
C10	0.62329 (8)	0.29990 (9)	0.34760 (12)	0.0309 (3)	
C11	0.61314 (9)	0.32625 (10)	0.22259 (13)	0.0346 (3)	
C12	0.61377 (12)	0.41579 (12)	0.19286 (16)	0.0496 (4)	
H12	0.6228	0.4586	0.2533	0.059*	
C13	0.60111 (15)	0.44164 (14)	0.07450 (18)	0.0645 (6)	
H13	0.601	0.5015	0.0551	0.077*	
C14	0.58865 (16)	0.37784 (16)	−0.01468 (18)	0.0660 (6)	
H14	0.5801	0.395	−0.0944	0.079*	
C15	0.58871 (13)	0.28936 (13)	0.01287 (15)	0.0524 (4)	
H15	0.5804	0.2473	−0.0483	0.063*	
C16	0.60098 (9)	0.26197 (11)	0.13089 (13)	0.0372 (3)	
N17	0.59641 (9)	0.17030 (9)	0.15649 (13)	0.0397 (3)	
H17A	0.5924 (14)	0.1410 (14)	0.092 (2)	0.053 (6)*	
H17B	0.6415 (14)	0.1544 (11)	0.2191 (19)	0.040 (5)*	
O18	0.5	0.02352 (11)	0.25	0.0668 (7)	
H18	0.5330 (14)	−0.0001 (16)	0.3054 (19)	0.066 (7)*	
N19	0.67858 (9)	−0.01854 (9)	0.48037 (12)	0.0434 (3)	
O20	0.60669 (8)	−0.05918 (8)	0.44950 (11)	0.0528 (3)	
O21	0.73467 (10)	−0.03031 (13)	0.58020 (13)	0.0808 (5)	
O22	0.69082 (10)	0.03590 (10)	0.40974 (15)	0.0712 (4)	

### Atomic displacement parameters (Å^2^)

**Table:**

	*U*^11^	*U*^22^	*U*^33^	*U*^12^	*U*^13^	*U*^23^
Zn1	0.03883 (14)	0.02325 (12)	0.03001 (13)	0	0.00485 (9)	0
N2	0.0331 (5)	0.0301 (6)	0.0296 (5)	−0.0014 (4)	0.0078 (4)	−0.0025 (4)
C3	0.0311 (6)	0.0359 (7)	0.0303 (6)	0.0021 (5)	0.0081 (5)	−0.0015 (5)
C4	0.0476 (9)	0.0412 (8)	0.0396 (8)	−0.0031 (6)	0.0120 (7)	0.0035 (6)
C5	0.0563 (10)	0.0629 (11)	0.0368 (8)	−0.0011 (8)	0.0153 (7)	0.0108 (8)
C6	0.0474 (9)	0.0708 (12)	0.0292 (7)	−0.0006 (8)	0.0102 (6)	−0.0037 (7)
C7	0.0373 (7)	0.0496 (9)	0.0343 (7)	−0.0013 (6)	0.0090 (6)	−0.0097 (6)
C8	0.0288 (6)	0.0392 (7)	0.0320 (7)	0.0017 (5)	0.0087 (5)	−0.0040 (5)
N9	0.0362 (6)	0.0332 (6)	0.0344 (6)	−0.0076 (5)	0.0114 (5)	−0.0069 (5)
C10	0.0281 (6)	0.0316 (7)	0.0326 (6)	0.0000 (5)	0.0102 (5)	−0.0045 (5)
C11	0.0344 (7)	0.0377 (7)	0.0328 (7)	−0.0055 (5)	0.0130 (5)	−0.0027 (5)
C12	0.0633 (11)	0.0425 (9)	0.0426 (8)	−0.0154 (8)	0.0177 (8)	−0.0028 (7)
C13	0.0902 (15)	0.0509 (11)	0.0520 (11)	−0.0227 (10)	0.0240 (10)	0.0093 (8)
C14	0.0855 (15)	0.0755 (14)	0.0402 (9)	−0.0255 (12)	0.0256 (9)	0.0052 (9)
C15	0.0620 (11)	0.0649 (11)	0.0364 (8)	−0.0171 (9)	0.0246 (7)	−0.0087 (8)
C16	0.0336 (7)	0.0448 (8)	0.0364 (7)	−0.0050 (6)	0.0162 (6)	−0.0060 (6)
N17	0.0429 (7)	0.0391 (7)	0.0367 (7)	0.0044 (5)	0.0129 (6)	−0.0102 (5)
O18	0.0787 (14)	0.0254 (8)	0.0592 (12)	0	−0.0243 (10)	0
N19	0.0440 (7)	0.0394 (7)	0.0416 (7)	−0.0037 (5)	0.0079 (5)	−0.0051 (5)
O20	0.0555 (7)	0.0511 (7)	0.0437 (6)	−0.0187 (6)	0.0066 (5)	0.0105 (5)
O21	0.0619 (9)	0.1084 (14)	0.0499 (8)	0.0073 (8)	−0.0093 (7)	−0.0063 (8)
O22	0.0671 (9)	0.0601 (9)	0.0866 (11)	−0.0203 (7)	0.0266 (8)	0.0165 (7)

### Geometric parameters (Å, º)

**Table:**

Zn1—O18	1.9479 (17)	N9—H9	0.74 (2)
Zn1—N2^i^	2.0421 (11)	C10—C11	1.467 (2)
Zn1—N2	2.0421 (11)	C11—C12	1.395 (2)
Zn1—N17^i^	2.2147 (14)	C11—C16	1.408 (2)
Zn1—N17	2.2147 (14)	C12—C13	1.383 (3)
N2—C10	1.3285 (18)	C12—H12	0.93
N2—C3	1.3908 (17)	C13—C14	1.381 (3)
C3—C4	1.390 (2)	C13—H13	0.93
C3—C8	1.396 (2)	C14—C15	1.372 (3)
C4—C5	1.382 (2)	C14—H14	0.93
C4—H4	0.93	C15—C16	1.387 (2)
C5—C6	1.401 (3)	C15—H15	0.93
C5—H5	0.93	C16—N17	1.422 (2)
C6—C7	1.375 (2)	N17—H17A	0.86 (2)
C6—H6	0.93	N17—H17B	0.87 (2)
C7—C8	1.395 (2)	O18—H18	0.77 (2)
C7—H7	0.93	N19—O21	1.2235 (18)
C8—N9	1.379 (2)	N19—O22	1.228 (2)
N9—C10	1.3518 (18)	N19—O20	1.2601 (17)
			
O18—Zn1—N2^i^	122.42 (3)	C8—N9—H9	127.4 (15)
O18—Zn1—N2	122.42 (3)	N2—C10—N9	111.48 (12)
N2^i^—Zn1—N2	115.17 (7)	N2—C10—C11	124.91 (12)
O18—Zn1—N17^i^	96.88 (4)	N9—C10—C11	123.58 (13)
N2^i^—Zn1—N17^i^	80.04 (5)	C12—C11—C16	119.21 (14)
N2—Zn1—N17^i^	92.55 (5)	C12—C11—C10	120.13 (14)
O18—Zn1—N17	96.88 (4)	C16—C11—C10	120.65 (14)
N2^i^—Zn1—N17	92.55 (5)	C13—C12—C11	120.70 (17)
N2—Zn1—N17	80.04 (5)	C13—C12—H12	119.6
N17^i^—Zn1—N17	166.24 (7)	C11—C12—H12	119.6
C10—N2—C3	106.20 (11)	C14—C13—C12	119.43 (18)
C10—N2—Zn1	120.54 (9)	C14—C13—H13	120.3
C3—N2—Zn1	130.45 (10)	C12—C13—H13	120.3
C4—C3—N2	130.43 (14)	C15—C14—C13	120.80 (17)
C4—C3—C8	120.91 (13)	C15—C14—H14	119.6
N2—C3—C8	108.66 (12)	C13—C14—H14	119.6
C5—C4—C3	117.13 (16)	C14—C15—C16	120.73 (17)
C5—C4—H4	121.4	C14—C15—H15	119.6
C3—C4—H4	121.4	C16—C15—H15	119.6
C4—C5—C6	121.54 (16)	C15—C16—C11	119.12 (15)
C4—C5—H5	119.2	C15—C16—N17	119.91 (15)
C6—C5—H5	119.2	C11—C16—N17	120.88 (14)
C7—C6—C5	121.85 (15)	C16—N17—Zn1	108.50 (9)
C7—C6—H6	119.1	C16—N17—H17A	107.9 (14)
C5—C6—H6	119.1	Zn1—N17—H17A	121.4 (14)
C6—C7—C8	116.50 (15)	C16—N17—H17B	110.7 (11)
C6—C7—H7	121.7	Zn1—N17—H17B	95.1 (13)
C8—C7—H7	121.7	H17A—N17—H17B	112.7 (18)
N9—C8—C7	132.26 (14)	Zn1—O18—H18	117.5 (18)
N9—C8—C3	105.73 (12)	O21—N19—O22	119.85 (16)
C7—C8—C3	122.00 (14)	O21—N19—O20	121.32 (16)
C10—N9—C8	107.93 (12)	O22—N19—O20	118.78 (14)
C10—N9—H9	123.6 (15)		

Symmetry code: (i) −*x*+1, *y*, −*z*+1/2.

### Hydrogen-bond geometry (Å, º)

**Table:**

*D*—H···*A*	*D*—H	H···*A*	*D*···*A*	*D*—H···*A*
N9—H9···O21^ii^	0.74 (2)	2.51 (2)	3.248 (2)	173 (2)
N9—H9···O22^ii^	0.74 (2)	2.37 (2)	2.944 (2)	134.7 (19)
N17—H17*A*···O20^iii^	0.86 (2)	2.14 (2)	2.9937 (17)	169 (2)
O18—H18···O20	0.77 (2)	1.92 (2)	2.6897 (14)	175 (2)
O18—H18···O22	0.77 (2)	2.50 (2)	3.0345 (16)	128 (2)

Symmetry codes: (ii) −*x*+3/2, −*y*+1/2, −*z*+1; (iii) *x*, −*y*, *z*−1/2.
