# Supplementary figures and images for: Crystal structure of aqua­bis­[2-(1H-benzimidazol-2-yl-κN 3)aniline-κN]zinc dinitrate
Source: Acta Crystallogr E Crystallogr Commun. 2015 Mar 14;71(Pt 4):m85–6. doi: 10.1107/S2056989015004636 (PMC4438813; doi:10.1107/S2056989015004636)

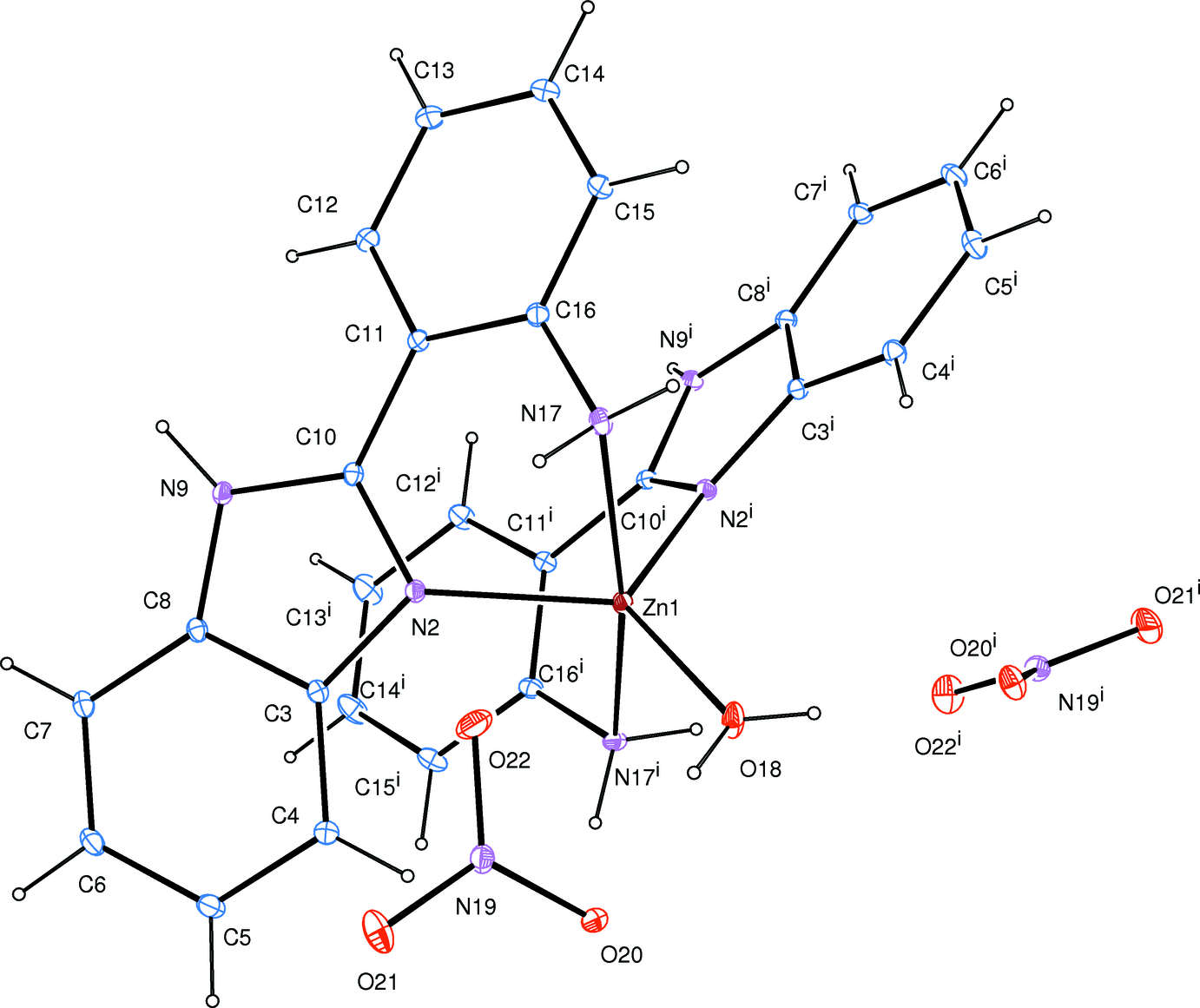

Supplement: Supplementary file 3 [file e-71-00m85-fig1.tif]

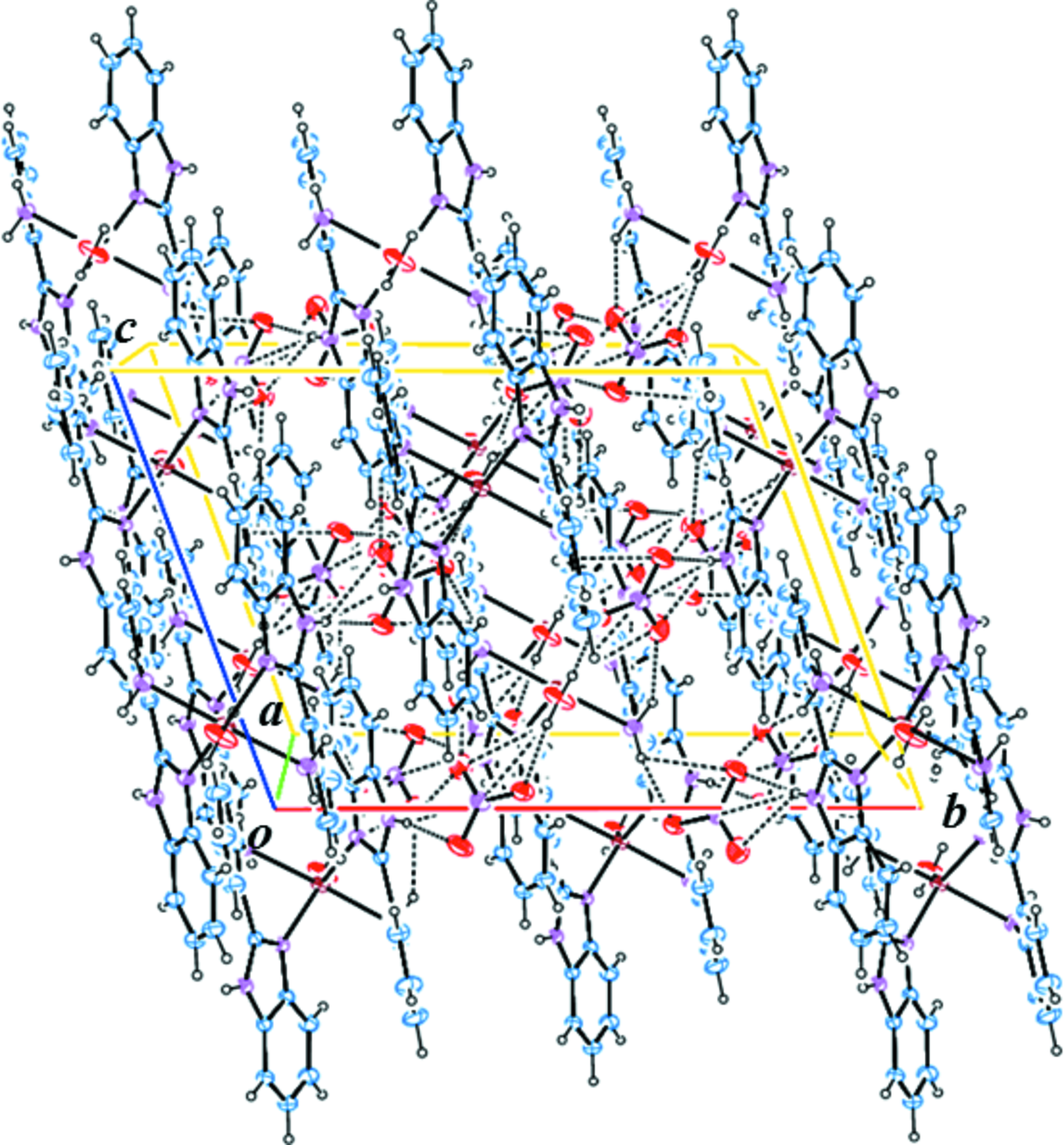

Supplement: Supplementary file 4 [file e-71-00m85-fig2.tif]
